# Development and implementation of the international AIDA network spondylarthritis registry

**DOI:** 10.3389/fmed.2025.1509357

**Published:** 2025-02-14

**Authors:** Valeria Caggiano, Antonio Vitale, Andrea Hinojosa-Azaola, Guillermo Arturo Guaracha-Basañez, Piero Ruscitti, Paola Cipriani, Samar Tharwat, Hanan M. Elberashi, Esraa E. Othman, Alessandro Conforti, Giancarlo Gimignani, Sukran Erten, Patrizia Barone, Maissa Thabet, Jurgen Sota, José Hernández-Rodríguez, Verónica Gómez-Caverzaschi, Gaafar Ragab, Amina Maher, Ezgi Deniz Batu, Perla Ayumi Kawakami-Campos, Jiram Torres-Ruiz, Carla Gaggiano, Abdurrahman Tufan, Hamit Kucuk, Henrique A. Mayrink Giardini, Guiga Ahmed, Mehmet Akif Eksin, Lampros Fotis, Azadeh Shariat Panahi, Stefano Gentileschi, Ibrahim A. Almaghlouth, Petros P. Sfikakis, George E. Fragoulis, Costanza Monterosso, Daniela Opris-Belinski, Soad Hashad, Nunzia Di Meglio, Cristian Sica, Bahar Özdemir Ulusoy, Alejandra de-la-Torre, Ewa Wiesik-Szewczyk, Katarzyna Rybak, Alberto Balistreri, Claudia Fabiani, Maria Antonietta Mazzei, Bruno Frediani, Luca Cantarini

**Affiliations:** ^1^Department of Medical Sciences, Surgery and Neurosciences, Research Center of Systemic Autoinflammatory Diseases and Behçet's Disease Clinic, University of Siena, Siena, Italy; ^2^Azienda Ospedaliero-Universitaria Senese [European Reference Network (ERN) for Rare Immunodeficiency, Autoinflammatory and Autoimmune Diseases (RITA) Center], Siena, Italy; ^3^Department of Immunology and Rheumatology, Instituto Nacional de Ciencias Médicas y Nutrición Salvador Zubirán, Mexico City, Mexico; ^4^Rheumatology Unit, Department of Biotechnological and Applied Clinical Sciences, University of L'Aquila, L'Aquila, Italy; ^5^Rheumatology and Immunology Unit, Internal Medicine Department, Mansoura University, Mansoura, Egypt; ^6^Department of Internal Medicine, Faculty of Medicine, Horus University, New Damietta, Egypt; ^7^Ospedale San Paolo di Civitavecchia, U.O. Medicina Generale, Rome, Italy; ^8^Department of Rheumatology, Faculty of Medicine Ankara City Hospital, Ankara Yıldırım Beyazıt University, Ankara, Türkiye; ^9^Pediatric Rheumatology Unit, Department of Integrated Maternal-Child and Reproduction Activity AOU "Policlinico-San Marco", Catania, Italy; ^10^Internal Medicine Department, Farhat Hached University Hospital, Faculty of Medicine of Sousse, University of Sousse, Sousse, Tunisia; ^11^Clinical Unit of Autoinflammatory Diseases, Department of Autoimmune Diseases, Institut d'Investigacions Biomèdiques August Pi I Sunyer (IDIBAPS), Hospital Clínic of Barcelona [European Reference Network (ERN) for Rare Immunodeficiency, Autoinflammatory and Autoimmune Diseases (RITA) Center], University of Barcelona, Barcelona, Spain; ^12^Rheumatology and Clinical Immunology Unit, Internal Medicine Department, Faculty of Medicine, Cairo University, Giza, Egypt; ^13^Faculty of Medicine, Newgiza University, 6th of October City, Egypt; ^14^Department of Pediatric Rheumatology, Faculty of Medicine, Hacettepe University, Ankara, Türkiye; ^15^Department of Ophthalmology, Instituto Nacional de Ciencias Médicas y Nutrición Salvador Zubirán, Mexico City, Mexico; ^16^Department of Internal Medicine, Division of Rheumatology, Gazi University Hospital, Ankara, Türkiye; ^17^Rheumatology Division, Faculdade de Medicina, Hospital das Clínicas, Universidade de São Paulo, São Paulo, Brazil; ^18^Clinic of Rheumatology, Ankara Gaziler Physical Therapy and Rehabilitation Training and Research Hospital, Ankara, Türkiye; ^19^Department of Pediatrics, Attikon General Hospital, National and Kapodistrian University of Athens, Athens, Greece; ^20^Rheumatology Unit, Department of Medicine, College of Medicine, King Saud University, Riyadh, Saudi Arabia; ^21^College of Medicine Research Center, College of Medicine, King Saud University, Riyadh, Saudi Arabia; ^22^Joint Academic Rheumatology Program, Medical School, National and Kapodistrian University of Athens, Athens, Greece; ^23^Department of Rheumatology, Università Vita-Salute San Raffaele, Milan, Italy; ^24^Rheumatology and Internal Medicine Department, Carol Davila University of Medicine and Pharmacy, Bucharest, Romania; ^25^Rheumatology Department Tripoli Children Hospital, Tripoli, Libya; ^26^Unit of Diagnostic Imaging, Department of Medical, Surgical and Neuro Sciences and of Radiological Sciences, University of Siena, Siena, Italy; ^27^Neuroscience Research Group (NEUROS), NeuroVitae Center, Escuela de Medicina y Ciencias de la Salud, Universidad del Rosario, Bogotá, Colombia; ^28^Department of Internal Medicine, Pneumonology, Allergology, Clinical Immunology and Rare Diseases, Military Institute of Medicine, National Research Institute, Warsaw, Poland; ^29^Bioengineering and Biomedical Data Science Lab, Department of Medical Biotechnologies, University of Siena, Siena, Italy; ^30^Ophthalmology Unit, Department of Medicine, Surgery and Neurosciences, University of Siena, Siena, Italy

**Keywords:** register, real life, treatment, autoinflamatory diseases, spondylarthritis, prognostic factor

## Abstract

During the last decade, spondyloarthritis (SpA) has increasingly been considered a disease at the crossroads between autoimmunity and autoinflammation. Some patients may even present with autoinflammatory-related manifestations, including fever, hidradenitis suppurativa, other neutrophilic dermatoses, and an unusually high increase in inflammatory markers. Therefore, a subgroup of SpA patients may be identified, and specific details about this cluster need to be investigated. In this regard, the AutoInflammatory Disease Alliance (AIDA) Network has developed a registry primarily aimed at better understanding the autoinflammatory aspects of SpA. The development of this Registry favors the systematic assessment of SpA through the lens of autoinflammation, giving a voice to patients with atypical presentations, and favoring a personalized treatment approach. By supporting research and facilitating the transfer of new evidence to clinical practice, this specific registry has the potential to significantly advance the field of rheumatology and enhance the lives of patients suffering from this complex and multifaceted disease.

## Introduction

Spondyloarthritis (SpA) encompasses a heterogeneous spectrum of chronic musculoskeletal diseases characterized by inflammation affecting the sacroiliac joints and the spine with or without peripheral joint involvement. Significant extra-articular manifestations, such as ocular, gastrointestinal, and cutaneous inflammatory disorders may be associate with SpA. In recent times, recurrent fever episodes have also been reported as a possible manifestation in SpA patients (1, 2). SpA includes a protean spectrum of clinical phenotypes, such as psoriatic arthropathy, enteropathic arthritis, and reactive arthritis. The pathogenesis of SpA is multifactorial, involving both autoimmune and autoinflammatory mechanisms. In recent years, research has increasingly emphasized the pivotal role of innate immunity in SpA, with growing evidence suggesting a crucial role of autoinflammatory processes (3). Specifically, the multiprotein complex NLRP3 inflammasome, known to be involved in various autoinflammatory diseases, has garnered interest for its role in SpA (4, 5). Indeed, this reflects the presence of specific groups of SpA patients characterized by peculiar clinical features, with autoinflammation appearing to prevail over the autoimmune component, both in terms of disease presentation and in relation to a less remarkable response to conventional therapy (6). In this perspective, the primary objective of this article is to outline the development of a registry dedicated to patients affected by SpA, focusing on evaluating the disease’s autoinflammatory aspects. Actually, this registry aims to gather comprehensive data about clinical presentation, laboratory findings, radiologic features, and treatment outcomes, especially when resembling other autoinflammatory conditions. Focusing on the autoinflammatory aspects of SpA, the registry seeks to enhance the understanding of less-known and rarer disease mechanisms and provides insights into new potential pathogenic aspects and therapeutic targets. Therefore, the development of this registry represents a critical initiative for the scientific and clinical community, serving as a robust platform to systematically study SpA through the lens of autoinflammation. The ultimate goal is to improve clinical management strategies and foster personalized medicine approaches based on the peculiar features that distinguish specific phenotypes from the majority of SpA patients. In this regard, the creation of a registry dedicated to these subgroups may enhance patient outcomes by navigating the complexities of this only apparently known rheumatic disease.

## Materials and methods

### Study design

The AIDA Registry presented in this work is an international, clinical, physician-driven, non-population-and electronic-based registry dedicated to patients diagnosed with SpA. Data collection includes a retrospective phase, for data gathered up to the time of enrollment in the Registry, and a prospective phase for data collected from the time of enrollment onwards. The prospective phase requires at least one follow-up visit per year, but data should also be collected whenever a change in the treatment strategy is required. The Registry is designed to collect demographic, clinical, laboratory, imaging, and treatment data starting from disease onset. This information will be derived from routine follow-up visits to adhere to the highest standard of care, without requiring additional data collection. Participation in this Project does not influence treatment choices or drug adjustments, which remain solely based on the patients’ disease activity, the literature evidences, and the physician’s clinical judgment. The Registry is open to all Centers involved in the management, diagnosis, and treatment of SpA and willing to participate in the project. Centers interested in participating can join the AIDA Network by contacting the AIDA Team at support@aidaregistry.org or by using the form available.^1^ The Network includes all clinical specialities, and neither the location nor the type of practice setting affects inclusion in the Project. Since the data entered by the Registry is typically part of standard SpA patient management, there are no associated costs or fees. Each Center must obtain approval from the local ethics committee to participate, and identify a Principal Investigator for local study coordination, as well as at least one Site Investigator appointed to manage documentation and guarantee data collection. Both the Principal Investigator and the Site Investigators will receive credentials to access the SpA Registry.

### Registry objectives

This International AIDA Registry for patients with SpA primarily aims to gather information from as many patients as possible, especially those with unusual presentations and those presenting with an autoinflammatory-like clinical picture. In this regard, the Registry aims to enable rapid and detailed understanding of the autoinflammatory subset of SpA, overcoming the delays typically associated with traditional clinical research, which often relies on limited study populations from single research centers. A large cohort is critical to obtain robust evidence to translate into daily clinical practice; data collection through an international on-line registry may quickly allow the achievement of this goal in terms of sample size and endpoints assessment. More in details, the specific objectives of the Registry are: (I) to characterize the wide spectrum of inflammatory manifestations and their frequency; (II) to identify rarer disease clusters, especially when facing with autoinflammatory-like clinical pictures; (III) to identify pathognomonic or at least specific features to facilitate diagnosis also in patients with atypical presentation of SpA; (IV) to contribute to the better understanding of the spectrum of autoinflammatory disorders, with consequent etiopathogenic and therapeutic perspectives; (V) to describe any long-term systemic complications that could specifically affect patients with rarer kinds of presentations; (VI) to identify prognostic factors to predict patients at higher risk of complications; (VII) to recognize predisposing factors and triggers associated with the onset and exacerbations of the disease, looking for a stratification of disease severity; (VIII) to describe treatment attempts performed over time, considering their overall efficacy and impacts on different aspects of the disease; (IX) to report the safety profile of treatment approaches in specific autoinflammatory SpA patients subgroups; (X) to identify the best treatment approach tailored to the patient’s features and disease characteristics, in order to search for a personalized treatment approach; (XI) to assess the influence of environmental factors and ethnic origin on the SpA phenotypes; (XII) to evaluate the impact of socioeconomic status on the access to healthcare facilities, specifically investigating the concepts of presenteism and absenteeism due to the presence and severity of the disease; (XIII) to monitor cardiovascular risk in SpA patients distinguishing between typical SpA patients and autoinflammatory SpA phenotypes; (XIV) to assess any differences in the autoinflammatory and treatment aspects between juvenile idiopathic arthritis with axial involvement and axial-SpA arising during adulthood; (XV) to monitor causes of death in SpA patients, especially in relation to the chronic exposure to systemic inflammation. Additionally, pioneering studies will be designed to address unmet needs identified during patient management, even those that will arise over time according to future acquisitions. Table 1 summarizes the primary and additional objectives of this Registry.

**Table 1:** Primary and additional objectives of AIDA network registry dedicated to patients affected by spondylarthritis.

Primary objectives	To gather extensive real-world data from a large cohort of patients enrolled on an international scale.
	To enable rapid and detailed understanding of the autoinflammatory subset of SpA
Additional objectives	To characterize the wide spectrum of inflammatory manifestations and their frequency in SpA
	To identify different disease subtypes with autoinflammatory-like clinical pictures
	To identify pathognomonic features to facilitate diagnosis
	To understand the full spectrum of autoinflammatory disorders, with consequent etiopathogenic and therapeutic perspectives
	To describe long-term systemic complications
	To identify prognostic factors to predict patients at higher risk of complications
	To recognize predisposing factors and triggers associated with the onset and exacerbations of the disease
	To describe treatment attempts, considering their overall efficacy and impacts on different aspects of the disease
	To report the safety profile of treatment approaches specific autoinflammatory SpA patients subgroups
	To identify the best treatment approach tailored to the patient’s features and disease characteristics
	To assess the influence of environmental factors and ethnic origin on the SpA phenotypes
	To evaluate the impact of socioeconomic status on access to healthcare and patient absenteeism due to the disease
	To monitor cardiovascular risk in SpA patients
	To assess differences between juvenile idiopathic arthritis with axial involvement and axial-SpA arising during adulthood
	Monitor causes of death in SpA patients, especially in relation to the chronic exposure to systemic inflammation
Ancillary objective	To design other pioneering studies to address unmet needs identified during patient management

SpA, spondylarthritis.

### Inclusion/exclusion criteria

The registry will include patients with SpA who fulfill the Assessment of SpondyloArthritis International Society (ASAS) criteria (7).

The patient has to give written and informed consent after a careful explanation of the Project regarding the objectives of the Registry, the lack of implications on both clinical management and treatment, the opportunity to withdraw the consent at any time, and the laws to comply with to guarantee patients’ privacy, anonymity and security of data. Actually, patients will be informed about the lack of consequences deriving from her/his will to participate or not in the study.

A legally authorized representative, who must observe the study requirements for the entire duration of the study or until the consent withdrawal, will have to act on the behalf of patients unable to provide their consent. The patient’s assent is essential for patients aged ≥12 years in any case.

Once inclusion criteria are fulfilled, no exclusion criteria or conditions may preclude the patients’ enrollment.

### Online data collection

The Research Electronic Data Capture (REDCap) instrument has been employed for data gathering and storing. REDCap is an electronic data collection tool developed at Vanderbilt University Medical Center (VUMC) and is currently hosted at Virginia Commonwealth University (Award Number UL1TR002649). The use of the REDCap platform is free for all members of the REDCap consortium, who benefit from technical support in exchange for using the tool. Actually, the consortium includes over 7,500 institutions from 159 countries across four continents (last access on 1 October 2024) (8).

The data are stored on servers of the University of Siena, Siena, Italy. Privacy is ensured for each Center’s data, with Principal and Site Investigators unable to access data collected in other Centers. The browser interface for data entry is provided entirely in English to minimize language barriers and facilitate international data collection. The retrospective assessment requires the collection of clinical and laboratory data related to the symptoms of the disease at onset, diagnosis, and enrollment into the Registry; clinical and laboratory data should be entered at the start of each treatment performed, at 3-months, 6-months, and 12-month visits, and at the last assessment while on treatment. On the other hand, prospective follow-up visits will be added for each visit conducted after enrollment in the AIDA Registry, at least every year and/or at any change in the treatment strategy, as for the introduction of new drugs and in case of posology changes. Socioeconomic data collected include variables designed to assess the impact of SpA on national healthcare systems (e.g., access to primary care physicians, specialist visits, laboratory examinations, imaging tests, emergency care, and hospitalization) and the workforce (e.g., absenteeism and presenteeism). The Investigators are responsible for the data they enter into the online Registry and must ensure the accuracy of the information; the Principal Investigator is required to verify the data’s accuracy. Online access through personal usernames and passwords guarantees the security of the patients’ information.

The investigators responsible for data collection will belong to reference centers for the clinical management of axial SpA patients and/or scientific research of axial SpA. Therefore, those entering the data must be adequately trained in the field of arthritis and, more specifically, spondyloarthritis. This requirement will enhance adequate uniformity of data collection at an international level.

### Ethics

The Ethics Committee of the Azienda Ospedaliero-Universitaria Senese, Siena, Italy (Ref. N. 14,951) granted the first national regulatory approval for the AIDA Project in June 2019. After this approval, numerous Centers with expertise in the diagnosis, clinical management, and treatment of autoinflammatory diseases have joined the AIDA Network from Europe, the Middle East, Africa, and the Americas. Patients’ information is managed in accordance with the EU General Data Protection Regulation (GDPR) on the processing of personal data and the protection of privacy (2016/679/EU) (9) and other national regulations referring to data protection. Regarding the patients’ voluntary informed consent, the AIDA registries comply with the recommendations of the Declaration of Helsinki. For minor patients aged 12 years and older who are not competent to provide consent, assent is required along with authorization from parents or legal guardians to permit the patient’s participation in the Project. Consent for processing data for statistical or research purposes may be withdrawn at any time by patients or Principal Investigators. In such cases, no further information will be collected, and patients have the right to request the complete erasure of all personal data already gathered in the Registry, if required and notified to the study Promoter (University of Siena). No financial remuneration is planned for patients or physicians participating in the study, and there will be no billing relationships with national health systems or insurance companies.

### Statistical analysis

The statistical analysis will be tailored to the specific types of studies that will be obtained from data collection and on the characteristics of the data used for the analysis. The analysis will incorporate fundamental principles of descriptive statistics, correlations among different groups, and comparisons between subgroups. Detailed statistical methodologies will be elucidated in upcoming papers derived from data sourced from the International AIDA SpA Registry. Principal Investigators and Site Investigators are encouraged to propose their study designs during regularly scheduled AIDA meetings. Data collected at individual centers may be analyzed independently by satellite Centers, leaving aside the need to acknowledge the REDCap tool and the AIDA Network in the papers deriving from data collected through the AIDA project. Conversely, data provided by different AIDA centers worldwide will be managed by statisticians and physicians affiliated with the Network, selected based on their specialized expertise from time to time.

## Results

**Figure 1:**
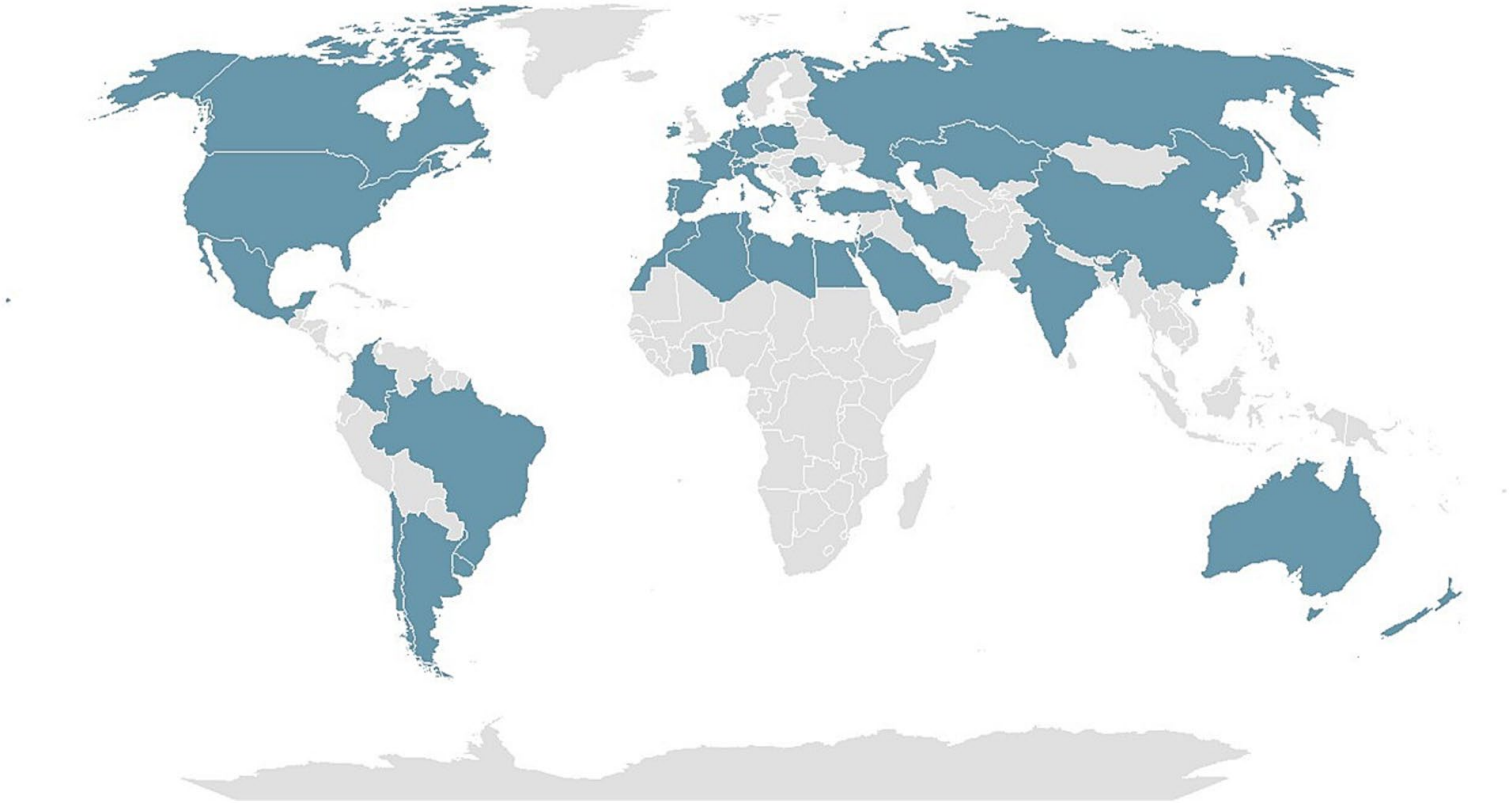
The map illustrates the global distribution of the autoinflammatory disease alliance (AIDA) network as of October 2024.

The development and launch of the International AIDA Network SpA Registry represent a pivotal achievement for the AIDA Project. This international registry dedicated to SpA is crucial for efficiently collecting real-world data on a global scale. Currently, a total of 251 Centers worldwide has participated in the AIDA project, involving 751 users, 251 principal investigators, 495 site investigators, two lead investigators, and three data managers. The project has already been registered on ClinicalTrials.gov (ID: NCT05200715). Figure 1 displays the worldwide distribution of the AIDA Network in October 2024.

### Registry development

When determining clinical variables to include in the Registry, the pivotal goal was to gather essential information to enhance the understanding of the autoinflammatory aspects of SpA, which are still inadequately explored at current. The Registry was meticulously designed to document the entire clinical and therapeutic history of enrolled patients within a relatively short timeframe. At current, the Registry encompasses 4,610 common data elements, each corresponding to a study variable, organized into 14 instruments. Among these instruments, 10 are designed for retrospective data collection, one is dedicated to prospective data collection, and three are intended for both retrospective data collection and specifying any clinical or treatment changes occurring from enrollment onward. Detailed information about these instruments, including their phases and the number of fields they encompass, is provided in Table 2. The common data elements include demographic, clinical, instrumental, laboratory, therapeutic, and other medical variables essential to fully characterize the disease course. Many of these elements are shared across other AIDA registries focused on various autoinflammatory and non-infectious ocular diseases, facilitating potential data integration among different Registries. To optimize efficiency, each variable is answered only when relevant to the patient’s clinical picture. This is facilitated by a branching mechanism that prompts the opening of answers only when necessary to complement previously provided information. Consequently, investigators encounter only a subset of the 4,610 variables tailored to the specifics of each patient’s clinical profile.

**Table 2:** The panel of instruments comprising the registry dedicated to patients affected by spondylarthritis (SpA) also includes the number of common data elements, along with the phase (i.e., retrospective/prospective) to which they should refer.

Instrument name	Retrospective/prospective phase	N fields
Demographics	Retrospective phase	11
Consents	Retrospective phase	5
Diagnostic data and family history	Retrospective phase	62
Features of SpA from the diagnosis to the enrollment	Retrospective phase	102
Clinical diagnostic scores and criteria	Retrospective phase	13
Radiologic features	Retrospective phase	32
Cardiovascular risk	Retrospective/Prospective phase	24
Treatments until the enrollment in the AIDA registry	Retrospective phase	1
Treatment with cDMARDs not associated to biologics	Retrospective phase	482
Treatment with small molecules not associated to biologics	Retrospective phase	1,112
Treatment with biologic agents	Retrospective phase	1,673
Fertility And Pregnancy	Retrospective/Prospective phase	15
Disease course and treatment during pregnancies	Retrospective/Prospective phase	82
Follow-up visits clinical manifestations and treatment	Prospective phase	982

AIDA, AutoInflammatory Disease Alliance; cDMARDs, conventional disease-modifying anti-rheumatic drugs; SpA, spondyloarthritis.

## Discussion

SpA is a complex and diverse group of chronic musculoskeletal diseases primarily affecting the sacroiliac joints and spine, potentially involving the peripheral joints. The disease spectrum includes distinct clinical phenotypes such as psoriatic arthropathy, enteropathic arthritis, and reactive arthritis, each presenting unique clinical and pathophysiological characteristics (10). Notably, SpA often extends beyond joint involvement to include significant extra-articular manifestations like ocular, gastrointestinal, and cutaneous inflammatory disorders, complicating its diagnosis and management (1).

Recent observations have identified recurrent fever episodes as an emerging manifestation in SpA patients, contributing to suggest that an autoinflammatory component is particularly relevant in the pathogenesis of at least a subset of SpA patients (2, 11). This aligns with the growing body of research highlighting the critical role of innate immunity and autoinflammatory mechanisms in SpA. The involvement of the NLRP3 inflammasome, a key player in various autoinflammatory diseases, has garnered particular interest, suggesting its potential contribution to SpA pathogenesis (6).

In addition, it is intriguing to note that both monogenic and multifactorial autoinflammatory diseases are frequently associated with SpA-like clinical features (12). This association underscores the intertwined nature of autoinflammatory mechanisms across a spectrum of rheumatologic conditions, further emphasizing the need for a comprehensive understanding of these processes.

Familial Mediterranean Fever, a genetic disorder caused by specific mutations of the *MEFV* gene leading to the dysregulation of the innate immune system, is the monogenic disease more frequently associated to SpA, which is reported in up to 13% of cases (13, 14). Of note, FMF-related *MEFV* variations have been found associated with ankylosing spondylitis (AS), and these variations might contribute to the pathogenesis of AS, especially in populations in which the prevalence of FMF is high (15).

Multifactorial autoinflammatory diseases, which arise from a combination of genetic predispositions and environmental triggers, often exhibit significant clinical overlap with SpA. Conditions like Behçet’s disease and Still’s disease may include axial or peripheral SpA, as well as enthesitis (13, 16, 17). These conditions illustrate the complex interplay between genetic factors and the immune system that contributes to the clinical manifestations seen in SpA.

The recognition of these overlaps is crucial to highlight the shared pathogenic pathways involving autoinflammation in both monogenic/multifactorial autoinflammatory diseases and SpA, at least in atypical and particularly inflammatory phenotypes. In this regard, NLRP3 inflammasome and subsequent IL-1β and IL-18 overproduction could represent only a fraction of the entire world of autoinflammation in SpA (6, 12, 13, 18, 19). Therefore, beyond the potential coexistence of specific monogenic autoinflammatory diseases, investigating the multifactorial autoinflammation in SpA is a crucial turning point for understanding the shared pathogenic mechanisms, with consequent impact on the diagnosis of atypical cases, therapeutic targets and personalized treatment strategies.

The extraordinary heterogeneity of SpA makes it necessary to investigate the various aspects of the disease in detail and specifically. Therefore, it is highly recommended to enroll one patient in different AIDA Network registries, if this patient suffers from ocular inflammatory involvement or other concomitant autoinflammatory disorders. For example, a SpA patient with uveitis or scleritis should also be enrolled in the International AIDA Network registries dedicated to uveitis or scleritis (20, 21), in order to properly investigate the ophthalmological characteristics of these patients. Likewise, patients with SpA and Behçet’s disease as well as patients with SpA and monogenic autoinflammatory diseases should be included in both the International AIDA Network Registry dedicated to SpA and in the International AIDA Network Registries for Behçet’s disease or autoinflammatory diseases, respectively (22, 23).

### Implications for clinical management and research

The clinical heterogeneity observed in SpA, including its possible overlap with monogenic and multifactorial autoinflammatory diseases, poses significant challenges for diagnosis and treatment. The varying responses to conventional therapies among different patient subgroups, particularly those with a prominent autoinflammatory component, underscore the necessity for personalized treatment approaches. For instance, a subtype of patients with systemic SpA symptoms could exhibit a poor response to TNF inhibitors and might benefit from therapies targeting the IL-1 pathway (2, 19).

Notably, the AIDA Network has been developed to facilitate comprehensive population-and non-population based data collection and to strengthen international collaboration by concentrating research efforts on global projects. In light of these insights, the establishment of an international registry mainly focused on the autoinflammatory aspects of SpA represents a significant advancement for research. The systematic collection and the following data analysis on clinical presentations, laboratory findings, radiologic features, and treatment outcomes will allow to elucidate the autoinflammatory component of SpA and to identify novel pathogenic pathways and therapeutic targets. Therefore, the primary reason leading to the development of this registry is to level out the lack of knowledge about autoinflammation in SpA, which remains less well-characterized compared to its autoimmune components. With this objective in mind, the registry aims to achieve the development of personalized medicine approaches tailored to specific SpA phenotypes. This is particularly critical at a time when it is crucial to ensure a personalized approach to the patient, as distinguishing between autoimmune and autoinflammatory processes can significantly improve treatment decisions and outcomes.

Similar to other AIDA registries (20–28), evaluating the socioeconomic impact of the disease on national healthcare systems, patients’ social roles, and employment outcomes constitutes a significant subject of analysis. Additional objectives will be established in response to emerging challenges in clinical practice and scientific research over the coming years. Indeed, the registry benefits from notable plasticity, being a tool capable of changing and adapting to evolving needs and future developments. The International AIDA SpA Registry is an invaluable resource for the scientific and clinical community, offering a robust platform for sharing large systematic studies and enabling the identification of rarer patterns that might otherwise remain concealed due to the reduced epidemiological impact.

Beyond the positive side of the Project, this registry shows the typical limitations of observational studies. Specifically, the main limitations could be related to the incompleteness of retrospective data and the clinical and therapeutic variability linked to different geographical contexts. In particular, access to diagnostic and therapeutic tools, as well as the different guidelines followed, may vary depending on the countries of origin of the participating centers. Notably, the completeness and accuracy of the data collected during the retrospective phase present significant challenges. The participation of centers worldwide entails different approaches and interpretative methods for the data entered in the registry. Therefore, these differing approaches may result in data that could not be fully consistent during statistical analysis. Additionally, the lack of a mandate to enroll all patients seen at AIDA Centers could introduce unintended selection bias. The process of enrolling patients into the Registry requires considerable time and attention, particularly when dealing with patients who have extensive medical histories due to complex clinical scenarios and multiple treatment approaches. Therefore, both investigators and patients enrolled must be sensitized as to provide their time for the study purposes. However, the same limitations of the study also represent an important resource, as the global reach of the project could enable the generation of results adjusted for the ethnicity and geographical region of the patients. Furthermore, it could allow for the identification and verification of potential differences in clinical presentation and therapeutic response among patients with axial SpA, as well as differences related to the primary and secondary objectives of the project.

All things considered, this Registry represents an invaluable tool to fully understand the new insights into the disease’s etiology and progression, ultimately leading to improved clinical management strategies and better patient care. Given the potential of the project and the ability to overcome its limitations thanks to the broad international reach of the registry, this article encourages the widest possible participation to achieve the registry’s objectives. In this regard, it is possible to contact the Network by visiting the webpage^2^ or by emailing the Network at support@aidanetwork.org.

## Conclusion

In conclusion, the development of a registry dedicated to SpA with an emphasis on autoinflammatory aspects addresses an unmet need that can further enrich our knowledge of SpA. In particular, it responds to the need for a nuanced understanding of different SpA clinical presentations, giving a voice to patients with atypical presentations for whom diagnosis can be complicated and delayed. Additionally, the standard treatment may not always be effective, precisely due to its non-personalized nature. By supporting research and facilitating the transfer of new evidence to clinical practice, the registry has the potential to significantly advance the field of rheumatology and enhance the lives of patients suffering from this complex and multifaceted disease. Additionally, the Registry may serve as a valuable resource for enrolling patients more easily in future randomized clinical trials.

## Data Availability

The original contributions presented in the study are included in the article/supplementary material, further inquiries can be directed to the corresponding authors.

## Footnotes

^1^ https://aidanetwork.org/en/aida

^2^ https://aidanetwork.org/it/aida/contacts
